# Nineteen nail dystrophy in an atopic background responsive to dupilumab: a case report

**DOI:** 10.3389/fmed.2026.1938551

**Published:** 2026-10-19

**Authors:** Yilan Zang, Xingmo Li, Huichao Li, Jinxu Qi, Qing Zhu, Guoqiang Zhang

**Affiliations:** 1 Department of Dermatology, The First Hospital of Hebei Medical University, Shijiazhuang, Hebei, China; 2 Subcenter of National Clinical Research Center for Skin and Immune Diseases, Shijiazhuang, Hebei, China; 3 Hebei Technical Innovation Center of Dermatology and Medical Cosmetology Technology, Shijiazhuang, Hebei, China

**Keywords:** atopy, dupilumab, nail changes, nail dystrophy, onychodystrophy

## Abstract

Onychodystrophy refers to abnormal nail structure or growth arising from disorders affecting the nail unit. Published reports of pediatric nail dystrophy occurring in an atopic background remain limited. We report a 7-year-old boy whose nail abnormalities were first noted at 3 weeks of age, progressively involved 19 of the 20 fingernails and toenails in the persistent absence of any eczematous lesions. Following an inadequate response to prior conventional therapies, the nail abnormalities showed marked improvement after 8 months of dupilumab therapy. In this patient, AD-associated nail involvement was considered a possible diagnosis after exclusion of common alternative causes, and the marked response to dupilumab further strengthened this possibility. This case highlights the importance of a broad differential diagnosis for pediatric nail dystrophy and suggests that dupilumab may be a therapeutic option when an atopic contribution is clinically suspected.

## Introduction

Onychodystrophy is a descriptive term referring to abnormal nail growth or morphology (1). It may result from disorders affecting different components of the nail unit, including the nail matrix, nail bed, nail plate, and nail folds. Trachyonychia is a characteristic pattern of nail-matrix involvement, presenting with rough, dull, longitudinally ridged or fissured nail plates, sometimes accompanied by pitting, koilonychia, or distal brittleness. It generally results from disruption of normal nail growth and keratinization caused by inflammatory, infectious, traumatic, mechanical, genetic, or systemic factors affecting the nail unit (2). Accordingly, onychodystrophy should be regarded as a nonspecific clinical manifestation and careful evaluation is required to identify the underlying cause. Pediatric nail disorders are relatively common, with reported prevalence estimates ranging from approximately 3% to 11% in earlier studies (3). Most nail disorders in children are benign and self-limited; however, some may represent the first manifestation of an underlying dermatologic, systemic, or genetic disease. Dermatologic conditions are particularly important in the differential diagnosis (3), including AD, psoriasis, lichen planus, alopecia areata, vitiligo, and onychomycosis.

AD is a chronic immune-mediated inflammatory disease that commonly begins in infancy or early childhood (4), characterized by relapsing pruritic eczematous eruptions (5). The disease demonstrates a strong genetic predisposition, frequently associated with a personal or family history of atopic diseases (6). It is widely accepted to be driven by complex interactions among genetic susceptibility, epidermal barrier dysfunction, immune dysregulation, microbial factors and environmental exposures. Nail changes in AD are frequently overlooked in routine clinical practice (7). A systematic review (8) reported that approximately 11% of patients with AD exhibited onychodystrophy. However, most reported cases occur in the context of cutaneous or periungual disease, and the diagnostic significance of isolated nail dystrophy in the persistent absence of eczematous lesions remains unclear.

Dupilumab is a fully human monoclonal antibody targeting interleukin-4 receptor alpha (IL-4R*α*) that inhibits signaling of IL-4/IL-13, thereby suppressing type 2 inflammation, improving epidermal barrier dysfunction and reducing pruritus (9). As the first biologic agent approved for moderate-to-severe AD, dupilumab has demonstrated significant efficacy in improving skin lesions, itch, and quality of life in patients with AD (10). Evidence for dupilumab-responsive nail dystrophy in patients with AD remains largely limited to individual reports and one pediatric case series (11–16). In the present case, AD-associated nail involvement was considered one possible diagnosis based on the patient's atopic history, laboratory findings, and the exclusion of alternative diagnoses. In view of the inadequate response to conventional therapy, dupilumab was administered after parental consent. After 32 weeks, the nail abnormalities improved markedly. This therapeutic response strengthened the possibility of a type 2 inflammatory contribution. We therefore report this pediatric case of nineteen nail dystrophy in an atopic background and review the relevant differential diagnoses and available evidence for targeted therapy, with particular attention to possible AD-associated nail involvement.

## Case report

The patient was a 7-year-old boy, with a height of 140 cm and a weight of 32 kg. He was brought to our outpatient clinic by his guardians in October 2025 with the chief complaint of “progressive dystrophy of the fingernails and toenails for 7 years.” His guardians reported that the nail abnormalities had first been noticed at 3 weeks of age and had gradually become more extensive over time. At presentation, no generalized erythema, eczematous eruptions, or lichenification were observed; the nail unit was the only clinically apparent site of disease. After disease onset, he was treated with desonide cream and 0.03% tacrolimus ointment, each applied once daily, but no satisfactory clinical response was achieved. Because of concerns regarding the potential adverse effects of systemic corticosteroids, his guardians declined oral corticosteroid therapy. The patient had allergic rhinitis, with no relevant family history or history of hereditary disease. He also denied any history of hepatitis or tuberculosis.

Physical examination revealed marked thickening, irregular roughening, and yellow-brown opaque discoloration of 19 of the 20 fingernails and toenails, with sparing of the right fifth toenail and no periungual erythema or soft-tissue swelling **(**Figures 1A–D**)**. Dermoscopy revealed irregular whitish patches, nail fissuring, and fine longitudinal grooves affecting the fingernails and toenails. The nail plates were thickened and showed yellow-brown opaque changes, with focal depressions in some areas **(**Figures 2I–K**)**. Laboratory tests revealed a significantly increased eosinophil percentage of 9.7% (reference range: 0%–0.9%), and an elevated absolute eosinophil count of 0.8 × 10^9^/L (reference range: 0–0.68 × 10^9^/L). Except for eosinophilia, the complete blood count, liver and renal function tests, electrolyte levels, and lipid profile were within normal ranges. Additional laboratory testing revealed a mildly elevated serum immunoglobulin E (IgE) level of 172.1 IU/mL (reference range: 0–60 IU/mL). Allergen testing showed sensitization to beef, mites, and mold. To exclude onychomycosis, direct microscopic examination and fungal culture of nail clippings obtained from the affected nails were performed three times at one-month intervals, with consistently negative results. Due to technical limitations, PAS staining of nail clippings and fungal PCR were not performed. His parents refused nail biopsy because of concern about possible permanent dystrophy. Considering the history of atopy, laboratory results, and exclusion of common alternative causes, AD-associated nail involvement was considered one possible diagnosis. At baseline, his scores of Children's Dermatology Life Quality Index (CDLQI) and Peak Pruritus Numerical Rating Scale (PPNRS) were 12 and 5, respectively.

**Figure 1:**
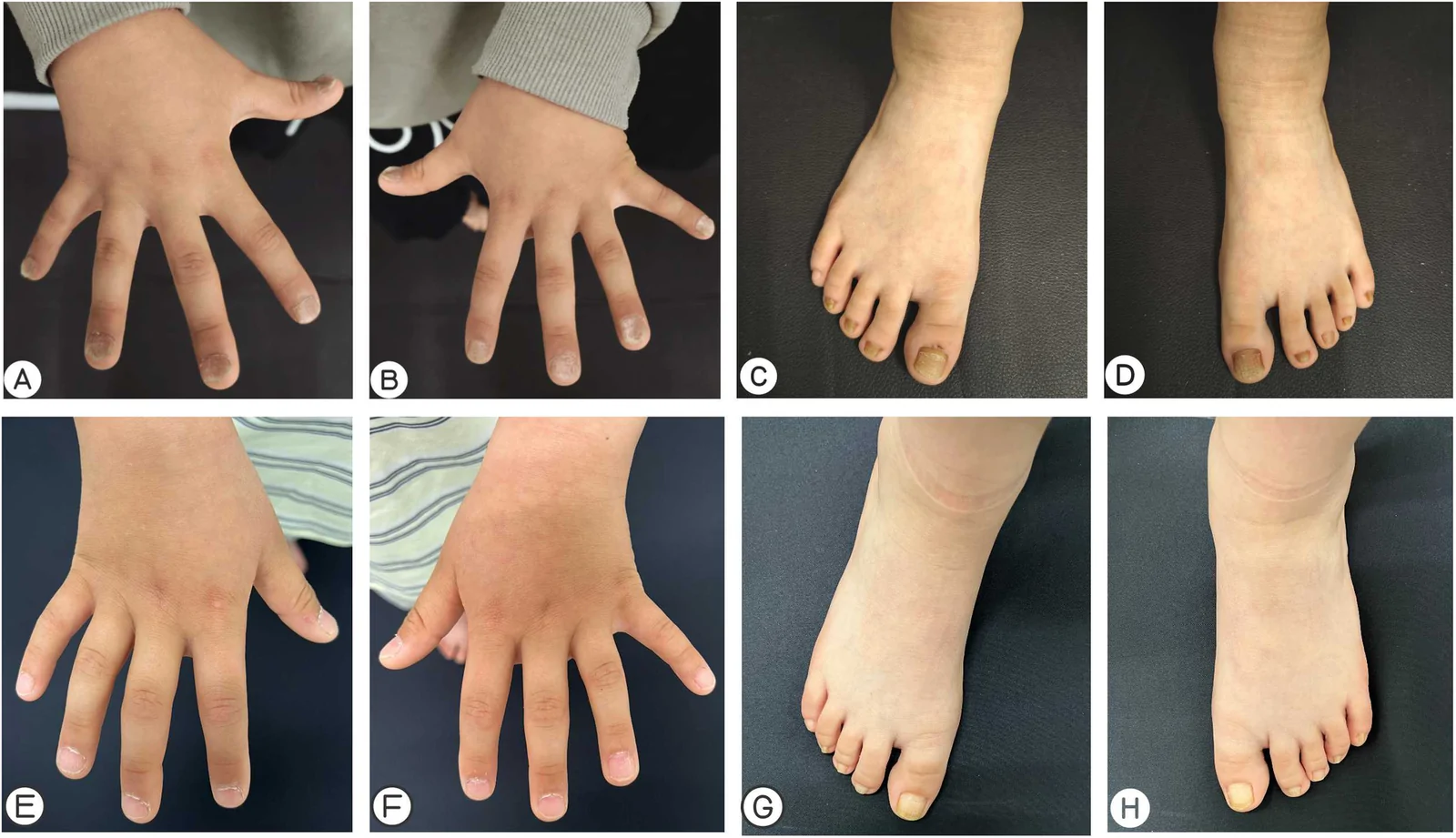
Clinical appearance of the affected fingernails and toenails before **(A–D)** and 32 weeks after treatment with dupilumab **(E–H)**. **(A–D)** With the exception of the right fifth toenail, all remaining nails exhibited marked thickening of the nail plates, irregular roughening of the nail surface and yellow-brown discoloration, without periungual erythema or soft-tissue swelling. **(E–H)** The affected nails showed near-complete restoration of normal morphology, thickness and color.

**Figure 2:**
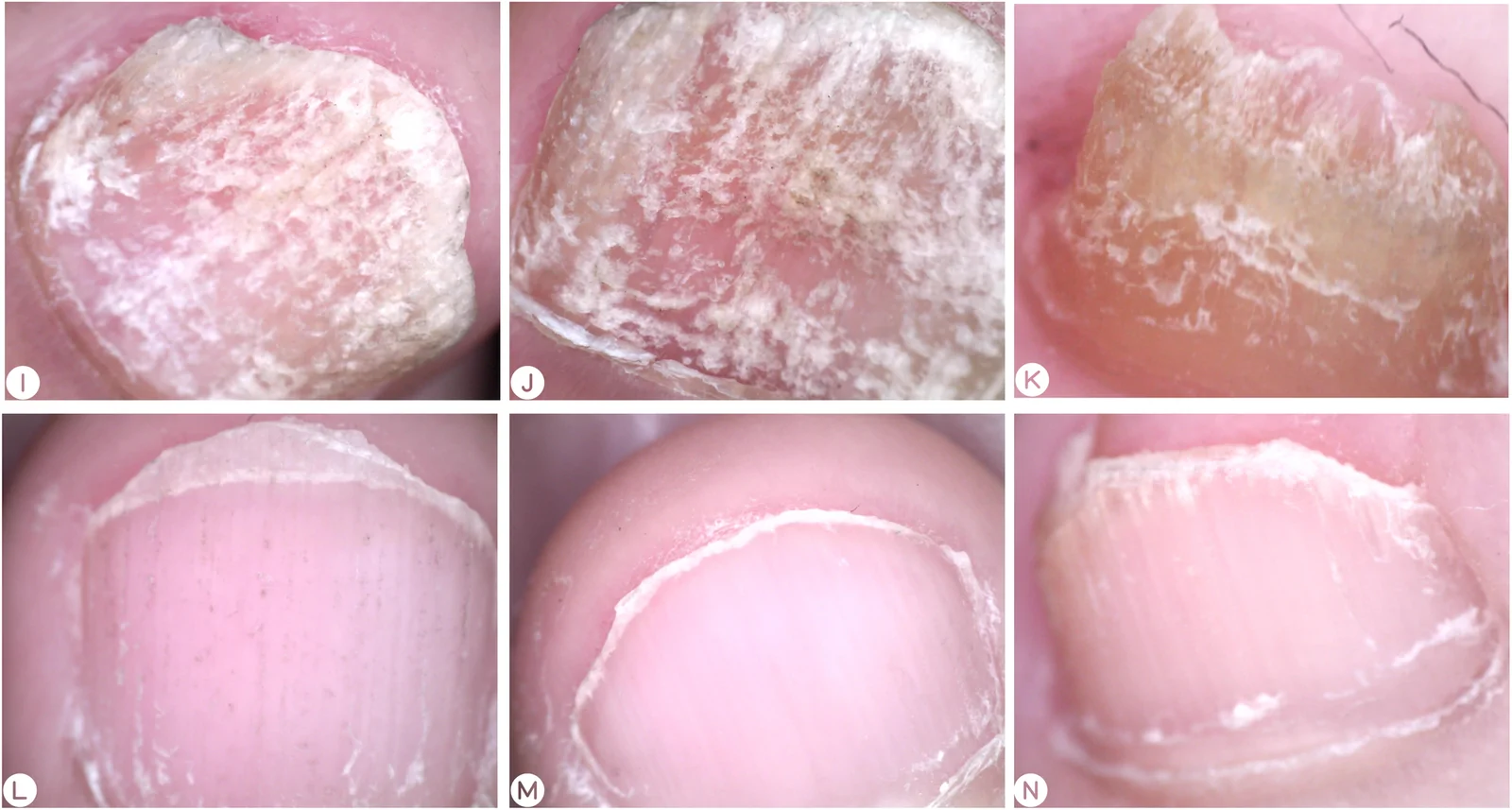
Dermoscopic appearance of the affected fingernails and toenails before and after treatment with dupilumab. **(I–K)** Dermoscopy showed diffuse nail plate thickening and surface roughening, yellow-brown discoloration, irregular whitish scaly patches, nail fissuring, fine longitudinal grooves, and focal nail plate depressions with partial destruction. **(L–N)** Dermoscopy showed marked improvement in nail plate morphology, with reduced surface roughness, discoloration, fissuring, and longitudinal ridging, together with progressive restoration of a smoother and more regular nail plate.

**Figure 3:**
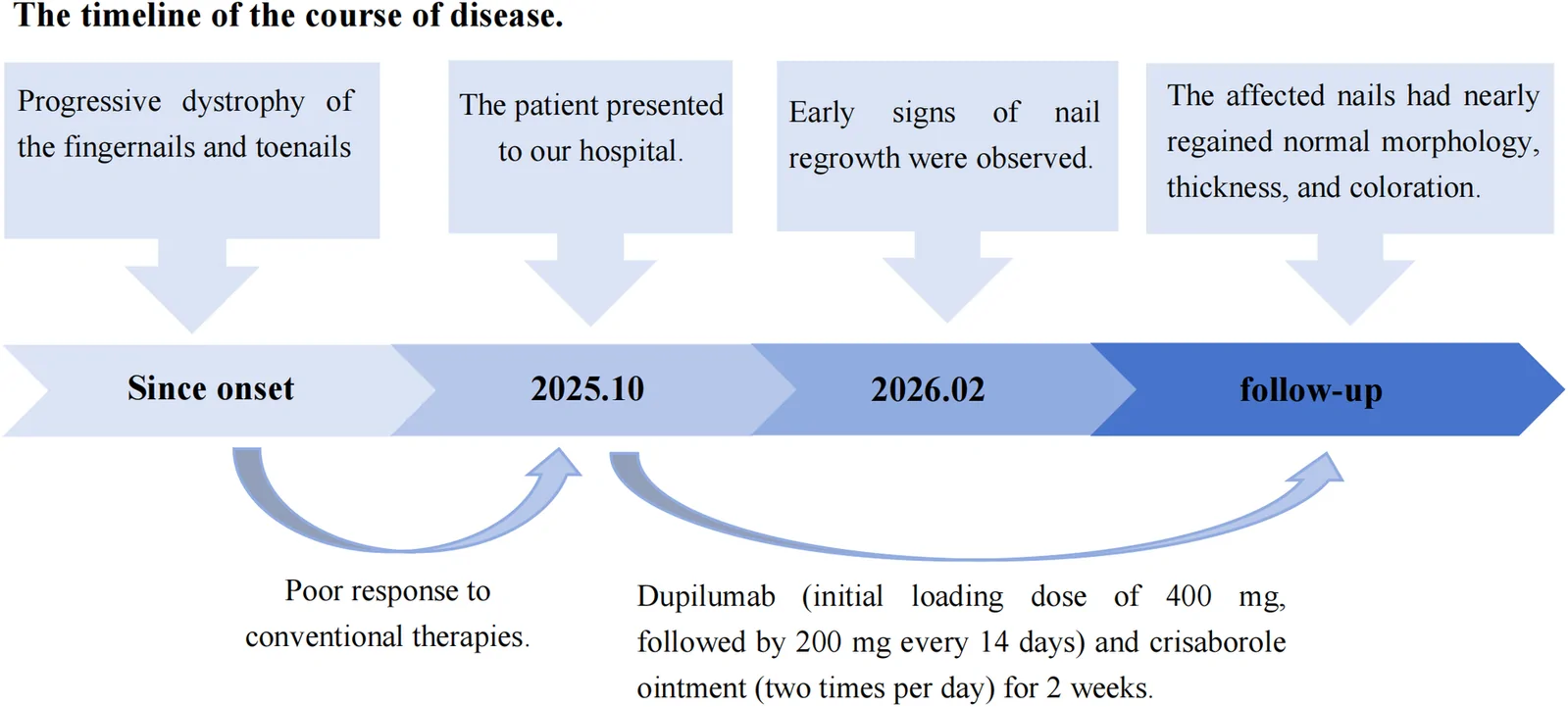
The timeline of the course of disease.

Given the inadequate response to previous conventional treatments, after obtaining parental consent, the patient received an initial loading dose of dupilumab 400 mg, followed by 200 mg every 2 weeks, in combination with topical crisaborole ointment applied twice daily to the affected lesions for 2 weeks. At the 16-week follow-up, early signs of nail regrowth were observed. At the 32-week follow-up, the affected nails had nearly regained normal morphology, thickness, and coloration **(**Figures 1E–H, 2L–N**)**, accompanied by marked reductions in CDLQI and PPNRS scores to 2 and 1. No adverse events were observed during dupilumab treatment, and the patient remained on maintenance therapy. To date, the patient has been followed for 8 months, and ongoing follow-up is being conducted to monitor treatment response and potential drug-related adverse events.

## Discussion

Nail dystrophy represents a clinical pattern of nail-unit involvement rather than a specific etiologic diagnosis, as similar changes may occur in psoriasis, lichen planus, alopecia areata, onychomycosis, vitiligo, AD-associated nail changes, and other nail disorders (17). Therefore, the diagnosis should rely on integration of the nail phenotype with associated cutaneous findings, clinical history, and exclusion of alternative causes. Nail psoriasis is a common manifestation of chronic plaque psoriasis and may occur independently or coexist with psoriasis affecting the scalp, extremities or trunk. The reported prevalence of nail psoriasis varies widely across studies, ranging from 10% to 80% (18), and is higher in patients with psoriatic arthritis (19). Thimble-like nail pitting, splinter hemorrhages and oil-drop discoloration are characteristic diagnostic features of nail psoriasis (20). Nail lichen planus (NLP) occurs in approximately 10% of patients with cutaneous lichen planus. It predominantly affects the fingernails and often presents with multiple, bilateral and symmetric nail lesions. The most common clinical findings in NLP are attributable to nail-matrix involvement: nail-plate thinning, longitudinal ridging, dorsal pterygium and distal splitting of the nail plate (21). On dermoscopy, linear nail-bed discoloration has been described as a distinctive feature of NLP (22). Both nail psoriasis and NLP may involve the nail matrix and nail bed; however, the severity of nail changes is not always associated with the severity of cutaneous disease. Nail involvement in alopecia areata (AA) is often associated with patchy or diffuse hair loss and is more common in children and in patients with severe disease. Most nail changes in AA are limited to nail matrix involvement, with pitting and trachyonychia as the most common clinical findings (21). Compared with nail psoriasis, AA-related pitting is typically smaller, shallower and more regularly distributed, often showing a grid-like pattern on the nail surface. Vitiligo-related nail abnormalities, such as longitudinal ridging or leukonychia, should be interpreted in the context of concomitant depigmented patches (23). Onychomycosis often presents with nail thickening, yellow-brown discoloration, subungual hyperkeratosis and onycholysis, and its diagnosis requires confirmation by mycological examination and fungal culture (24).

Nail abnormalities have also been reported in patients with AD. These alterations may occur in association with chronic periungual or hand eczema, although nail involvement is not necessarily proportional to the severity of cutaneous AD. The reported nail abnormalities include nail matrix changes such as Beau's lines or transverse grooves, nail pitting, trachyonychia, longitudinal ridging, leukonychia, onychomadesis, onychoschizia and melanonychia; nail bed changes such as onycholysis, koilonychia, brachyonychia and nail discoloration; and periungual manifestations such as chronic paronychia, periungual eczema and hangnails (8). Dermoscopic findings are generally nonspecific and may include thickened or rough nail plates, longitudinal ridging, onychoschizia, focal depressions, splinter hemorrhages, red lunulae and periungual scaling (7). Therefore, in patients with extensive nail dystrophy, a history of AD and other atopic manifestations, together with current or previous eczematous lesions involving the hands or periungual skin, may support AD-associated nail involvement. The most distinctive feature of this case was extensive nail-matrix involvement in the absence of generalized or periungual eczema. The patient's atopic history and laboratory evidence of type 2 inflammation suggested a possible contribution of an atopic inflammatory background to the nail changes. After excluding other common causes of nail dystrophy, AD-related nail involvement was therefore retained as one possible explanation. The marked improvement following dupilumab treatment further strengthened this possibility.

Given the patient's atopic background, an atopy-related mechanism was considered as one possible explanation for the extensive nail dystrophy. Atopic conditions are characterized by a complex interplay between epithelial barrier dysfunction and immune dysregulation, in which type 2 inflammation driven by Th2 cells and cytokines such as IL-4, IL-13 and IL-31 plays a central role (9). Dupilumab blocks IL-4R*α*-mediated IL-4/IL-13 signaling, thereby suppressing type 2 inflammation and reducing the clinical manifestations. Recent reports suggest that periungual eczema and pruritus may precede progressive nail changes, indicating that chronic inflammation around the nail may serve as an initiating factor of nail dystrophy in individuals with an atopic background. In patients with periungual eczema, the lesions may impair the local skin barrier and create a persistent Th2-dominant inflammatory microenvironment around the nail folds. This inflammation may subsequently affect the nail matrix and nail bed, thereby disturbing normal nail plate formation and leading to nail dystrophy (12). Wang et al. suggested that pruritus-driven scratching may mechanically injure the nail unit, while Chng and Yew noted that soaps and detergents can further impair the periungual barrier (8, 16). These factors may interact with each other, sustaining nail-unit injury and delaying nail recovery. Relief of pruritus may provide an additional benefit by reducing repetitive scratching and secondary matrix trauma (14). However, these mechanisms cannot fully explain the present case because periungual inflammation was not clinically evident. In this patient, topical tacrolimus and desonide cream failed to halt the progression of the nail dystrophy, whereas healthy nail regrowth became evident after 16 weeks of dupilumab treatment. This marked therapeutic response strengthens the possibility that subclinical inflammatory involvement of the nail unit may likewise be driven predominantly by type 2 inflammation, with IL-4/IL-13 acting as key mediators. However, in the absence of direct histopathological or molecular evidence from the nail unit, this proposed mechanism remains speculative.

We reviewed the literature on dupilumab-treated nail dystrophy in patients with AD. This evidence remains limited to case reports and a systematic review. We conducted a narrative literature search of PubMed from database inception to June 2026 using the terms “atopic dermatitis”, “dupilumab”, “nail dystrophy”, “onychopathy”, “nail changes” and related terms. A total of six published reports describing dupilumab treatment in AD patients with nail involvement were identified, including five adult single-case reports (11–15) and one pediatric case series (16), for a total of 10 patients. The clinical characteristics and outcomes of these patients are summarized in Table 1. Among these cases, one patient showed an unusual course: the nail abnormalities worsened after dupilumab initiation but gradually improved with continued treatment (12). The authors considered that this change did not necessarily represent a true adverse drug reaction to dupilumab. Moreover, similar nail-related adverse events have not been reported in clinical trials of dupilumab (25). Therefore, clinicians should not interpret early worsening of nail abnormalities alone as treatment failure or discontinue dupilumab solely on this basis.

**Table 1:** Reported cases of nail dystrophy in patients With atopic dermatitis treated With dupilumab.

**Authors**		**Age (years)/ sex**	**Duration of AD (years)**	**Skin lesion at baseline**	**Nail changes**	**Previous treatment**	**Dupilumab treatment duration**	**Outcome of nail changes**
Giura et al. (14)		55/Female	Developed in childhood	Severe AD exacerbation	Median canaliform nail dystrophy of Heller of the right thumbnail: a central longitudinal split with an inverted fir-tree pattern. No other fingernails or toenails involved.	Topical and systemic steroids or ciclosporin.	600 mg initial dose, then 300 mg every 2 weeks.	Month 4: complete remission of the median canaliform nail dystrophy.
Zubek & Vesely (12)		60s/Female	>40	Extensive eczematous plaques on the head, neck, chest, back, legs, arms, and hands.	Before dupilumab: chronic paronychia of both thumbnails. At month 3: bilateral thumbnail dystrophy with hyperkeratosis, periungual scaling, cuticle detachment, and onycholysis; right-sided fragility/koilonychia and left-sided Beau's lines with splitting.	Not reported.	Continued 300 mg every 14 days.	Month 3: nail findings improved dramatically with residual longitudinal ridging, improving distal dystrophy, and less prominent horizontal ridging. Month 15: significant improvement; only very mild chronic paronychia with transverse ridges, longitudinal ridging, and nail pits remained.
Navarro-Triviño et al. (15)		61/Male	>40	Extensive eczematous plaques on chest, back, legs, and arms, including both hands and fingernails.	Chronic fingernail onychodystrophy with onychorrhexis, hyponychial hyperkeratosis, scaling of the lateral nail folds and eponychium, and loss of cuticle-to-nail-plate adhesion. Toenails had no relevant changes.	Methotrexate, cyclosporin, alitretinoin, etanercept, and adalimumab.	Not reported.	Month 3: all fingernails showed improvement confined to their proximal halves.
Li et al (13).		40/Female	3	Only periungual erythema and edema.	Twenty-nail dystrophy involving all fingernails and toenails: trachyonychia, roughness, and dullness.	Antihistamine, topical halometasone cream, pimecrolimus ointment, and oral corticosteroids.	Single 600 mg subcutaneous starting dose, followed by 300 mg every 14 days for 5 months; then 300 mg every 3 weeks.	Month 2: healthy new nail growth. Month 5: near-complete recovery, except for thumbnails.
Wang et al. (16)	Patient 1	4/Female	0.5	Scattered erythematous papules on the limbs.	Onychomadesis and leukonychia affecting fingernails and toenails.	Moisturizer and topical corticosteroid cream.	300 mg every 4 weeks.	Week 12: abnormal nails were largely replaced by healthy nails. Week 30: the clinical response remained stable.
	Patient 2	6/Male	3	Scattered erythema, papules, nodules, and hyperpigmentation on trunk, limbs, hands, and feet; some areas had erosion and crusting.	Beau's line, chronic paronychia, pitting, hyperkeratosis and erythema of the hyponychium, and loss of cuticle-to-nail-plate adhesion.	Oral cetirizine and topical corticosteroid creams.	600 mg initial dose, then 300 mg every 4 weeks.	Week 24: nail changes had essentially returned to normal.
	Patient 3	7/Male	4	Widespread erythema, papules, plaques, erosion, and crusting over the body.	Multiple transverse ridges along the midline of the left thumbnail plate and onycholysis on the surface of the right thumbnail.		600 mg initial dose, then 300 mg every 4 weeks.	Week 12: affected nails had almost completely returned to normal.
	Patient 4	4/Female	3	Generalized erythema, papules, plaques, erosion, and crusting on the trunk and limbs.	Trachyonychia, onycholysis, hyponychial hyperkeratosis, and chronic toenail paronychia.		300 mg every 4 weeks.	Week 12: paronychia almost resolved and affected fingernails grew out distally with gradual normalization. Week 24: nail lesions remained well controlled.
	Patient 5	8/Male	1	Scattered erythema and papules with erosions and crusts over the body.	Onychomadesis and proximal trachyonychia of fingernail plates; Beau's line of toenails.	Conventional antihistamines and topical corticosteroids.	600 mg initial dose, then 300 mg every 4 weeks.	Week 12: nails partly recovered; mild Beau's line and pitting remained.
Zhao et al. (11)		57/Female	10	Prior generalized eczematous eruptions on the extremities had resolved; marked periungual inflammation was present.	Five fingernails showed thickened, rough, yellow-brown dystrophic plates with periungual erythema and edema; toenails were normal.	Systemic corticosteroids; topical emollients; topical naftifine-ketoconazole cream and oral itraconazole.	600 mg loading dose followed by 300 mg subcutaneous injections every 2 weeks.	Week 12: periungual inflammation almost completely resolved; the nails became thinner and less opaque. Week 16: periungual erythema fully resolved and proximal new plates smoother/more translucent. Week 28: complete nail plate reconstruction. Week 52: largely normal morphology, no recurrence; only mild residual roughness reported.

Early adult reports showed that improvement in AD-associated nail dystrophy is typically delayed relative to the response of eczema and pruritus. This delay may reflect the time required for normal nail growth rather than treatment failure (26). Therefore, follow-up should be extended to cover at least one complete nail growth cycle in these patients. Wang et al. (16) provided the first pediatric case series, comprising five children aged 4–8 years. All five patients demonstrated marked clinical improvement after dupilumab. These findings add preliminary efficacy and safety data on dupilumab use in children with AD-associated nail changes. Together with the favorable response observed in the present case, these findings suggest that dupilumab may be a therapeutic option for nail dystrophy in patients with an atopic background. Nevertheless, conclusions remain constrained by small samples, heterogeneous follow-up, non-standardized outcome assessment, and limited histopathological confirmation. Larger prospective studies are needed to evaluate the efficacy and durability of targeted therapies for extensive nail dystrophy.

## Conclusion

In conclusion, this case describes a child with early-onset, extensive nail dystrophy in the persistent absence of eczematous skin lesions. The differential diagnosis included inflammatory, infectious, hereditary, and idiopathic causes; AD-associated nail involvement was considered one possibility based on the atopic history, laboratory findings, exclusion of common alternative causes, and marked response to dupilumab. The therapeutic response strengthens this possibility but does not establish a definitive diagnosis. Dupilumab may represent a potential therapeutic option in patients with inadequate response to conventional therapies. Further studies are needed to clarify disease mechanisms, define standardized outcome measures, and determine the efficacy and safety of targeted therapies in well-characterized nail-dystrophy phenotypes.

## Data Availability

The original contributions presented in the study are included in the article. Further inquiries can be directed to the corresponding authors.
